# Mortality among persons receiving tuberculosis treatment in Itezhi-Tezhi District of Zambia: A retrospective cohort study

**DOI:** 10.1371/journal.pgph.0001234

**Published:** 2023-02-22

**Authors:** Eutra Chaaba, Josphat Bwembya, Eness Nyambe, Ramya Kumar, Ibou Thior, Kaminsa Seraphine, Gershom Chongwe, Vimbai Makwambeni, Victoria Musonda, Pauline Kasese-Chanda, Alwyn Mwinga

**Affiliations:** 1 Ministry of Health, Itezhi-Tezhi District Health Office, Itezhi-Tezhi, Zambia; 2 USAID Eradicate TB Project, PATH, Lusaka, Zambia; 3 Zambart, Lusaka, Zambia; 4 PATH, Seattle, Washington, United States of America; 5 Tropical Diseases Research Centre, Ndola, Zambia; 6 USAID, Lusaka, Zambia; Zuckerberg San Francisco General Hospital and Trauma Center, UNITED STATES

## Abstract

Itezhi-Tezhi District in southern Zambia has been reporting tuberculosis (TB) mortality rates that are fourfold higher than the national average of six percent. We conducted a retrospective cohort study to establish the demographic and clinical characteristics associated with mortality among persons under treatment for TB in Itezhi-Tezhi District, as well as the likely causes and time to death. We reviewed medical records for persons with TB registered in 19 public health facilities in Itezhi-Tezhi District between January 2015 and December 2018. Of the 506 persons with TB registered in the study period, 426 were included in the analysis. Of these, 71 (16.7%) died before completing treatment. The overall mortality rate was 31.8 per 1,000 person-months of observation. Most of the deaths (53 [74.7%]) occurred in the first month of treatment (median: 16 days; interquartile range: 5–52 days). In a multivariate Cox regression model, type of TB was found to be an independent predictor of mortality while on TB treatment. The risk of dying was more than twice higher for persons with clinically diagnosed PTB compared to those with bacteriologically confirmed PTB (adjusted hazard ratio = 2.2, 95% CI: 1.4–3.6). In a sub-analysis of persons with clinically diagnosed PTB, persons with TB who were on a community-based DOT plan were more than twice more likely to die compared to those on facility-based DOT plan (adjusted hazard ratio = 2.21, 95% CI: 1.1–4.8). Common likely causes of death were pulmonary TB disease (66.0%), anemia (12.8%), cardiac failure (4.3%), pneumocystis jiroveci pneumonia (4.3%), and gastroenteritis (4.2%). These findings show that most deaths occurred during the first month of treatment. Clinical evaluation at initiation of anti-TB treatment and during follow-up care, especially in persons with clinically diagnosed PTB, should include screening and treatment of other conditions.

## Introduction

Tuberculosis (TB) is among the top ten causes of death worldwide and the leading cause of mortality ranking above human immunodeficiency virus (HIV)/acquired immunodeficiency syndrome (AIDS) [1]. In 2021, an estimated 1.3 million TB-related deaths occurred globally among people not infected with HIV, and 214,000 deaths were among those coinfected with HIV [1]. Zambia is among the high TB-burden countries in southern Africa that have recorded an impressive reduction in incidence rate in the last decade, from 759/100,000 population in 2000 to 361/100,000 in 2017 [2]. However, TB still remains among the top ten causes of mortality in Zambia [2]. The TB mortality rate among people living with HIV and those not infected with HIV in Zambia stands at 74/100,000 and 30/100,000, respectively [2].

Itezhi-Tezhi District in southern Zambia has been reporting TB mortality rates that are above the national average of 6% [3]. Reports from the District Health Office show that between 2015 and 2018, the average TB mortality rate in Itezhi-Tezhi was 22% [4]. There is limited information on the likely causes, characteristics, and predictors for TB-related mortality in Itezhi-Tezhi District.

Results from studies conducted in other settings identified history of smoking, hepatitis, malnutrition, diabetes mellitus, previous TB treatment, anemia, and alcohol and drug abuse as some of the factors associated with mortality among persons with TB [5]. Other studies identified liver cirrhosis and bacterial pneumonia as major causes of death in persons with TB, as well as old age, repeating anti-TB treatment, having a clinical diagnosis, and having an unknown HIV status [6]. Persons diagnosed with extra-pulmonary TB (EPTB) have also been found to be more likely to die than those with pulmonary TB (PTB) [7]. Persons coinfected with TB and HIV who do not receive cotrimoxazole presumptive therapy (CPT) are also more likely to die [7]. The majority of the TB-related deaths in resource-limited settings have been reported to occur in the first two months of treatment [6, 8] and have been attributed to delayed diagnosis or undiagnosed comorbidities [6, 8].

Without a clear understanding of the likely causes of death among persons with TB and associated risk factors, authorities in Itezhi-Tezhi District would not have the information needed to design and implement effective corrective measures. This study sought to describe mortality among persons with TB in Itezhi-Tezhi District by establishing time to death, predictors, and likely causes of death.

## Materials and methods

### Study design

This study was designed as a retrospective cohort study. Medical records of persons diagnosed with TB in Itezhi-Tezhi District between January 2015 and December 2018 were reviewed to determine treatment outcomes. For patients with a treatment outcome of ‘death,’ characteristics from their files were compiled for analysis. The study collected data on likely causes of death and time to death, following anti-TB treatment initiation, where available.

### Study sites

Itezhi-Tezhi District is situated in the Southern Province of Zambia. The district has 19 public health facilities servicing a population of 90,134 people [4]. There are two diagnostic facilities with microscopy—one also has an Xpert *Mycobacterium tuberculosis* (MTB)/rifampin (RIF) platform (which was introduced in 2018)—and 17 non-diagnostic facilities that provide anti-TB treatment, which we refer to as ‘treatment facilities.’ All health facilities have standard recording tools for presumptive TB (i.e., presumptive TB registers), and the two diagnostic facilities have laboratory registers.

Persons with presumed TB are entered into the presumptive TB registers, and their sputum is collected and sent to the laboratory at one of the two diagnostic facilities for examination. At the laboratory, patient details including results of the sputum examination are recorded in laboratory registers. Feedback on results is provided by sending back laboratory request forms and via phone calls, in some instances. Persons diagnosed with TB have their treatment information documented in a TB treatment register, and they are treated with a combination of isoniazid, rifampicin, ethambutol and pyrazinamide for a period of 6 to 12 months based on national guidelines—i.e., 6 months is the recommended period for treatment of susceptible TB disease and 12 months for severe forms, such as TB meningitis [9]. Treatment outcomes are recorded in TB treatment registers. For persons who die within a health facility, the cause of death is recorded in the patient’s file and a death certificate bearing the cause of death is issued.

### Study population and sampling methods

This study purposely sampled all 19 public health facilities that provide TB services in Itezhi-Tezhi District: 2 diagnostic facilities and 17 non-diagnostic facilities/treatment facilities. All persons diagnosed with TB between January 2015 and December 2018 in these facilities were included in the study. Persons that transferred from other districts were excluded because their treatment outcomes were expected to be reported back to the diagnosing districts.

### Data collection

In April 2020, this study collected secondary medical record data on the characteristics of persons diagnosed with TB from January 2015 to December 2018, their treatment outcomes, and likely causes of death, using a paper-based data extraction tool.

The data collection process started by compiling a list of persons diagnosed with TB at the two diagnostic facilities in the review period. This information was collected from the laboratory registers for persons with bacteriologically confirmed TB (i.e., TB involving the lungs or other parts of body with biological specimen positive by Xpert MTB/RIF, smear microscopy, or culture) and from treatment registers for persons with clinically diagnosed PTB. Date of diagnosis was also captured from the same registers.

Patient clinical characteristics were collected from TB treatment registers and categorized as follows: sex (male or female); access to GeneXpert (yes or no); type of TB patient (new patient or retreatment patient); type of TB (bacteriologically confirmed PTB, clinically diagnosed PTB, or EPTB); HIV status (positive, negative, or unknown); antiretroviral therapy (ART) treatment (on ART or not on ART); and directly observed therapy (DOT) plan (facility-based or community-based); (See detailed definitions in S1 File). Treatment outcomes were also captured from TB treatment registers and categorized according to the Ministry of Health’s national TB treatment guidelines [9] (Table 1). Dates of treatment initiation and treatment outcome, including date of death, were captured from TB treatment registers and death certificates. For patients that died at home and the actual date of death was not documented, the date of last visit to the health facility was used as a proxy for the date of death. Data on cause of death were collected from inpatient files and death certificates.

**Table 1:** Definition of treatment outcomes.

Treatment outcome	Definition
Cured	A TB patient with bacteriologically confirmed TB at the beginning of treatment, who was smear- or culture-negative in the last month of treatment and on at least one previous occasion.
Treatment completed	A TB patient who completed treatment without evidence of failure, but with no record to show that sputum smear or culture results in the last month of treatment, either because tests were not done or because results were unavailable, and on at least one previous occasion was negative.
Treatment failure	A TB patient whose sputum smear or culture is positive at month five or later during treatment.
Died	A TB patient who dies for any reason before starting or during the course of treatment.
Lost to follow-up (LTFU)	A TB patient who did not start treatment or whose treatment was interrupted for two consecutive months or more.
Not evaluated	A person with TB for whom no treatment outcome is assigned. ‘Not evaluated’ includes persons with TB who transferred out to another treatment unit in another district as well as those for whom treeatment outcome is unknown to the reporting unit.

Source: Tuberculosis manual. Ministry of Health, Lusaka (2017).

In Zambian rural settings like Itezhi-Tezhi, causes of death for people that die at home are not documented at health facilities or any government agency, nor is a verbal autopsy performed. Therefore, our data collection on likely causes of death was limited to persons with TB that died from health facilities.

### Data analysis

Data from the paper-based extraction tool were entered into SPSS Version 22, where it was cleaned by checking duplicate records, missing information, and inconsistencies by generating frequency tables in SPSS. It was then exported to Stata Version 13 for analysis. Categorical variables (sex, treatment facility, patient HIV status, type of patient, and type of TB, as well as commenced on CPT, ART for people living with HIV, and DOT plan) including the treatment outcome variable and cause of death were summarized using percentages that were presented in the form of frequency tables. Medians were used to summarize continuous variables such as age. Time to treatment initiation was calculated by subtracting the date of TB diagnosis from the date of treatment initiation. Time to treatment outcome, including time to death, was calculated by subtracting the date of treatment initiation from date of treatment outcome to find the total days of follow-up. We then divided total days of follow-up by 30 to obtain the total months of follow-up for all subjects in the study.

Kaplan-Meier survival curves for different patient categories were generated and compared using the log-rank test at 5% level of significance. The association between patient characteristics and the risk of death was assessed using a Cox proportional hazard regression model. The outcome variable was binary defined as died or did not die (cured, treatment completed, treatment failure, lost to follow-up or not evaluated). All cases with TB treatment outcomes other than death (our event of interest) were right censored at the end of the follow-up period (6 months). On bivariate analysis, variables with a p-value of less than 0.20 were selected as candidates for multivariate analysis. Predictors of TB-related mortality previously described in the literature including sex, age, HIV status, and patient type, were also added to the final model. Cox proportional hazards assumption was tested using Schoenfeld’s global test at 0.05 level of significance. Variables with a p-value of less than 0.20 at multivariate analysis were considered as statistically significant predictors of mortality among persons with TB. We conducted a sensitivity analysis to assess the robustness of our results by running an identical multivariable model that recorded all persons LTFU as having died.

### Ethics statement

Ethical approval for this study was granted by the University of Zambia Biomedical Research Ethics Committee (Ref #: 787–2020). Since our study relied on secondary data from patient registers and files, the requirement for participant consent was waived.

The reporting of this study conforms to the Strengthening the Reporting of Observational Studies in Epidemiology (STROBE) guidelines (S2 File).

## Results

### Description of the cohort

Out of a total of 506 persons with TB who were registered at 19 health facilities between January 2015 to December 2018, 80 were excluded because of missing information such as treatment outcomes (n = 50), type of TB (n = 19), DOT plan (n = 7), and date of treatment initiation (n = 4). A total of 426 TB cases were analyzed by this study, with an overall follow-up time of 2235 person months.

The highest number of persons with TB (130) was recorded in 2016, while the least (71) was recorded in 2018. The highest proportion (46.7%) of persons with TB was in the 30–44 years age group. The median age was 35 years (interquartile range [IQR]: 28–44 years). The majority (61.3%) of these persons with TB were male. Only 25 (5.8%) of the persons with TB accessed a facility with GeneXpert. Two hundred and twenty-seven (53.3%) persons with TB had bacteriologically confirmed PTB, 155 (36.4%) had clinically diagnosed PTB, and 44 (10.3%) had EPTB. Most of the persons with TB (359 [84.3%]) were newly diagnosed, with retreatments (relapses and treatment after default) accounting for 15.7% (67). The DOT plan for the majority (366 [85.9%]) of the persons with TB was facility-based (Table 2). The median time between TB diagnosis and treatment initiation was zero days, representing same day treatment initiation (IQR: 0). All persons with TB had a known HIV status. The majority (65.5% [279]) were people living with HIV, out of which 256 (91.8%) were on ART. Of those coinfected with TB and HIV, the majority (257 [92.1%]) were on CPT (Table 2).

**Table 2:** Demographic and clinical characteristics of persons with TB in Itezhi-Tezhi District (2015–2018), n = 426.

Category	Number of persons who died (%)	Number of persons who did not die (%)	Total	P-value
Total	71 (16.7)	355 (83.3)	426	
Sex				
Male	42 (16.1)	219 (83.9)	261	0.69
Female	29 (17.6)	136 (82.4)	165	
Age group (years)				
0–14	2 (8.7)	21 (91.3)	23	0.48^†^
15–29	14 (13.2)	92 (86.8)	106	
30–44	36 (18.1)	163 (81.9)	199	
45–59	14 (22.2)	49 (77.8)	63	
60 and older	5 (14.3)	30 (85.7)	35	
Accessed facility with GeneXpert				
No	69 (17.2)	332 (82.8)	401	0.40^†^
Yes	2 (8.0)	23 (92.0)	25	
Type of TB				
Bacteriologically confirmed PTB	28 (12.3)	199 (87.7)	227	0.012^†^
Clinically diagnosed PTB	37 (23.9)	118 (76.1)	155	
EPTB	6 (13.6)	38 (86.4)	44	
Type of patient				
Retreatments	9 (13.4)	58 (86.6)	67	0.44
New cases	62 (17.3)	297 (82.7)	359	
DOT plan				
Facility-based	56 (15.3)	310 (84.7)	366	0.06
Community-base	15(25.0)	45 (75.0)	60	
Year of treatment				
2015	14 (14.1)	85 (85.9)	99	0.63
2016	26 (20.0)	104 (80.0)	130	
2017	19 (15.1)	107 (84.9)	126	
2018	12 (16.9)	59 (83.1)	71	
HIV status				
Negative	22 (15.0)	125 (85.0)	147	0.49
Positive	49 (17.6)	230 (82.4)	279	
Unknown	0(0)	0(0)	0	
On ART (n = 279)				
No	5 (21.7)	18 (78.3)	23	0.57^†^
Yes	44 (17.2)	212 (82.8)	256	
On CPT (n = 279)				
No	3 (13.6)	19 (86.4)	22	0.61^†^
Yes	46 (17.9)	211 (82.1)	257	

ART, antiretroviral therapy; CPT, cotrimoxazole presumptive therapy; DOT, directly observed therapy; EPTB, extra-pulmonary tuberculosis; HIV, human immunodeficiency virus; PTB, pulmonary tuberculosis; TB, tuberculosis.

Percentages represent ‘row’ rather than ‘column’ totals

^†^Fisher’s exact test

From the total 426 persons with TB, 189 (44.4%) were cured and 158 (37.1%) completed treatment; therefore, the treatment success rate was 81.5%. The number of persons with TB who died while on treatment was 71 (16.7%) and 7 (1.6%) were lost to follow-up, while treatment failed in 1 (0.2%) patient. The mortality rate was 14.4% for persons who started TB on treatment in 2015, which then increased to 20.0% in 2016, dropped down to 15.1% in 2017, before increasing once again to 16.9% in 2018 (Table 3). The overall mortality rate was 31.8 per 1,000 person months of observation (95% CI: 25.2/1,000–40.1/1,000).

**Table 3:** Treatment outcomes for persons with TB in Itezhi-Tezhi District by year of treatment initiation (2015–2018).

Treatment outcome	Number of persons with TB (%)				Total (%)
	**2015**	**2016**	**2017**	**2018**	
Cured	17 (17.2)	46 (35.4)	77 (61.1)	49 (69.0)	189 (44.4)
Treatment completed	65 (65.7)	53 (40.8)	30 (23.8)	10 (14.1)	158 (37.1)
Treatment failure	-	1 (0.8)	-	-	1 (0.2)
Lost to follow-up	3 (3.0)	4 (3.1)	-	-	7 (1.6)
Died	14 (14.1)	26 (20.0)	19 (15.1)	12 (16.9)	71 (16.7)
Total	99 (100)	130 (100)	126 (100)	71 (100)	426 (100)

### Time to death

The majority of the deaths (74.7%) occurred in the first month of TB treatment (Table 4). The median time to death during TB treatment was 16 days (IQR: 5–52 days). Survival time seen from Kaplan-Meier plots for different patient categories showed that type of patient was significantly associated with survival. Persons with clinically diagnosed PTB died at a faster rate compared to those with bacteriologically confirmed PTB (p-value = 0.010, log-rank test) (S1 Fig).

**Table 4:** Time to death for persons with TB treated in Itezhi-Tezhi District (2015–2018), n = 426.

Time on treatment (in months)	Number of persons on treatment	Number of persons who died (%)	Incidence rate (per 1000 person months)	95% CI
1	426	53 (74.7)	981	750–1,285
2	372	7 (9.9)	500	238–1,049
3	365	2 (2.8)	167	42–667
4	361	4 (5.6)	250	75–94
5	357	3 (4.2)	200	65–620
6	354	2 (2.8)	1	0–4

### Characteristics associated with TB mortality in Itezhi-Tezhi District

In a multivariate Cox regression model, type of TB was found to be an independent predictor of mortality while on TB treatment (Table 5). The risk of dying was more than twice higher for persons with clinically diagnosed PTB compared to those with bacteriologically confirmed PTB (adjusted hazard ratio = 2.2, 95% CI: 1.4–3.6) (Table 5). In a sub-analysis of persons with clinically diagnosed PTB (n = 155), DOT plan was significantly associated with mortality. Persons with TB who were on a community-based DOT plan were more than twice more likely to die compared to those on facility-based DOT plan (adjusted hazard ratio = 2.2, 95% CI: 1.1–4.8) (S1 Table). A sensitivity analysis in which all all persons LTFU were re-coded as having died also showed that persons with TB on community-based DOT plan were almost twice more likely to die compared to those on a facility-based DOT plan (adjusted hazard ratio = 1.85, 95% CI: 1.1–3.2) (S2 Table).

**Table 5:** Characteristics associated with TB mortality in Itezhi-Tezhi District (2015–2018), n = 426.

**Category**	**cHR (95% CI)**	**aHR (95% CI)**
	**Univeriate**	**Multivariate**
Sex**		
Male	1	1
Female	1.1 (0.7–1.8)	1.1 (0.7–1.8)
Age (years)**		
0–14	0.5 (0.1–2.0)	0.5 (0.1–1.9)
15–29	0.7 (0.4–1.4)	0.73 (0.4–1.4)
30–44	1	1
45–59	1.3 (0.7–2.3)	1.1 (0.6–2.1)
60 and older	0.8 (0.3–2.0)	0.6 (0.3–1.7)
Type of TB		
Bacteriologically confirmed PTB	1	1
Clinically diagnosed PTB	2.0 (1.2–3.3)	2.0 (1.2–3.3)
EPTB	1.1 (0.4–2.7)	1.2 (0.5–3.0)
Type of patient**		
Retreatment	1	1
New	1.3 (0.7–2.6)	1.4 (0.7–2.8)
DOT plan		
Facility-based	1	1
Community-based	1.7 (1.0–3.0)	1.7 (1.0–3.1)
HIV status**		
Negative	1	1
Positive	1.2 (0.7–1.9) 05	1.1 (0.6–1.8)

aHR, adjusted hazard ratio; ART, antiretroviral therapy; cHR, crude hazard ratio; CI, confidence interval; DOT, directly observed therapy; EPTB, extra-pulmonary tuberculosis; HIV, human immunodeficiency virus; PTB, pulmonary tuberculosis; TB, tuberculosis.

*Significant at 0.20 level of significance.

**Added to the final model on the basis of being a clinical predictor of mortality.

### Likely causes of death among persons with TB in Itezhi-Tezhi District (2015–2018)

Of the 71 persons with TB who died, 47 (66.2%) had likely causes of death recorded at the health facility, with the major likely cause being PTB disease itself (66%) and anemia (12.8%). Other likely causes of death included cardiac failure (4.3%), pneumocystis jiroveci pneumonia (4.3%), and gastroenteritis (4.2%). Hypotension, liver cirrhosis, meningitis, and cardiovascular accidents each accounted for 2.1% of the deaths (Table 6).

**Table 6:** Likely causes of death among persons with TB in Itezhi-Tezhi District as recorded on death certificate (2015–2018), n = 47.

Category	Number	Percent
PTB	31	66.0
Anemia	6	12.8
Cardiac failure	2	4.3
Pneumocystis jiroveci pneumonia	2	4.3
Chronic gastroenteritis	1	2.1
Cardiovascular accident	1	2.1
Gastroenteritis	1	2.1
Hypotension	1	2.1
Liver cirrhosis	1	2.1
TB meningitis	1	2.1
**Total**	**47**	**100**

## Discussion

This study sought to describe mortality among persons with TB in Itezhi-Tezhi District by establishing time to death, predictors, and likely causes of death. The TB mortality rate in our study was 16.7%, higher than the national average of 6%. The figure was also higher than the 9% reported from a study conducted in Lusaka, the capital of Zambia [10]. Relatively higher TB mortality rates in rural areas compared to urban parts of Zambia were also reported in the Southern Province of the country [11].

We however noted that mortality rates for the years 2017 and 2018 reported by this study were less than what was reported through routinely collected data (15.1% vs. 23% and 16.9% vs. 22%, respectively). This finding might be an indication of weaknesses in routine data management that needs further investigation by the district health management team. Routine data verification activities and onsite staff mentorship in data quality assessment, recording, and reporting would facilitate the availability of more accurate data in this setting.

The majority of the deaths in our study occurred in the intensive phase of TB treatment (i.e., during the first month). This finding is similar to what was reported in Malawi and Ethiopia [8, 12]. Higher mortality in the intensive phase of TB treatment may be attributed to delayed presentation at the health facilities on the part of persons with TB [12], delayed diagnosis, and/or delayed treatment initiation. Due to inadequate documentation, our study could not collect data on time to care-seeking and time to diagnosis. However, delays in seeking care and diagnosis may be true for Itezhi-Tezhi District which only has two TB diagnostic facilities. Persons with TB from across the district are referred to the two facilities for diagnosis. Long distances to reach the nearest diagnostic facility and/or associated transport costs may cause a patient to delay seeking health services and only present at the facility when the disease is at an advanced stage. Delays in health-seeking in sub-Saharan Africa may also be due to prior consultation of a traditional healer [13]. Community awareness, knowledge, and demand for TB services needs to be strengthened. Further research is required to understand the health-seeking behavior and pathway to care of persons with TB in Itezhi-Tezhi District. However, both the median and IQR for time to treatment initiation for this study were zero, suggesting that delayed TB treatment initiation may not be a problem for Itezhi-Tezhi District.

Our study also found that persons with clinically diagnosed PTB had a higher risk of dying compared to those with bacteriologically confirmed PTB. Similar findings were reported in Ethiopia [14], South Africa [6], and Nigeria [13]. Harries et al. argued that in resource-limited settings, death among persons with clinically diagnosed PTB may be due to incorrect diagnosis as differentiating PTB from other conditions such as pneumocystis jirovecii pneumonia can be very difficult in a routine clinical setting [8]. Comprehensive evaluation of persons with TB prior to treatment initiation and close monitoring of response to treatment may help detect undiagnosed conditions. Continuous mentorship can also help build service providers’ capacity and skills to differentiate TB from other related conditions.

This study also found that persons with TB on a community-based DOT plan were more likely to die compared to those that were on facility-based DOT plan. This finding could in part be attributed to the reduced number of active community-based TB treatment supporters, which followed the departure of a key implementing partner in the district that funded community-level TB activities, including patient tracking and adherence counseling.

Our study collected data on likely causes of death among persons with TB through a review of death certificates and patient files. The findings show that deaths among persons with TB may also be due to other underlying conditions such as anemia, cardiac failure, pneumocystis jiroveci pneumonia, and gastroenteritis. This finding is similar to results from a study conducted in Iran [15]. Likely causes of death unrelated to TB accounted for 34% in our study and was higher than the 26% that was reported in the study from Iran [15] but lower than the 98% reported in China [16]. Although our findings on cause of death are limited by the lack of autopsy reports, which are considered the gold standard for reporting cause of death [16], establishing a system for comprehensive screening and treatment of underlying conditions can help prevent death among persons with TB in Itezhi-Tezhi District.

The major strengths of our study include the availability of date of events in TB treatment registers, allowing for performance of time to event (survival) analysis. This information is usually lacking in most program-level data. We also were able to collect information on the likely causes of death among persons with TB from a review of death certificates and patient files, as opposed to a limited conclusion based on demographic and clinical characteristics associated with death.

Our findings should however be viewed in the context of a number of limitations that broadly relate to use of programmatic data whose accuracy and completeness may not be fully guaranteed. Firstly, specific likely causes of death among persons with TB may not have been accurately determined due to the lack of autopsy results. Secondly, records on the likely cause of death for almost half of the persons with TB who died could not be found. These are people who may have died at home. Therefore, our findings on likely causes of death may not be an accurate representation of causes of death among persons with TB.

Further, data extraction for this study was limited to variables that are routinely captured in TB treatment registers per national guidelines. Hence, other clinical and socioeconomic factors which may also contribute to death—such as time to TB diagnosis, time to HIV diagnosis, time to ART initiation, history of smoking, and adherence to treatment—were not included in this study. In addition, we were unable to collect elapsed time between onset of clinical manifestations and TB diagnosis. In this regard, our findings may not be applicable to settings where these factors are important. Our findings may, however, be generalisable to rural parts of Zambia where conditions are largely similar to those prevailing in Itezhi-Itezhi District.

## Conclusion and recommendations

The study found that mortality during TB treatment was extremely common in Zambia’s Itezhi-Tezhi District and that the majority of TB-related deaths occurred in the first month of treatment. Notably, compared to persons with bacteriologically confirmed PTB, those with clinically diagnosed PTB were two-times more likely to die. Based on these findings, we recommend improved availability of GeneXpert for bacteriological TB diagnosis coupled with strengthening of the DOTs program to monitor TB disease progression and response to treatment, especially during the intensive phase

## Supporting information

S1 File Detailed definition variables used in study on mortality among persons receiving tuberculosis treatment in Itezhi-Tezhi District of Zambia. (DOCX)

S2 File Completed STROBE checklist. (DOCX)

S1 Table Sub analysis of characteristics associated with TB mortality in Itezhi-Tezhi District–among person with clinically diagnosed PTB (2015–2018), n = 155. (DOCX)

S2 Table Sensitivity analysis of characteristics associated with TB mortality in Itezhi-Tezhi District recoding all persons LTFU as having died (2015–2018), n = 426. (DOCX)

S1 Fig Kaplan-Meier survival curves by type of TB for persons with TB in Itezhi-Tezhi District (2015–2018), n = 426. (DOCX)
