# Supplementary material for: Mortality among persons receiving tuberculosis treatment in Itezhi-Tezhi District of Zambia: A retrospective cohort study
Source: PLOS Glob Public Health. 2023 Feb 22;3(2):e0001234. doi: 10.1371/journal.pgph.0001234 (PMC10021721; doi:10.1371/journal.pgph.0001234)
Supplement: S1 File — (DOCX) [file pgph.0001234.s001.docx]

**S1 File: Detailed definition variables used in study on Mortality among persons receiving tuberculosis treatment in Itezhi-Tezhi District of Zambia**

| **Variable** | **Categories** | **Definition** |
| --- | --- | --- |
| Type of TB | Bacteriologically confirmed pulmonary tuberculosis | TB involving the lungs with biological specimen positive by GeneXpert, smear microscopy or, culture |
|  | Clinically diagnosed pulmonary tuberculosis | TB involving the lungs but without laboratory confirmation. This definition includes cases diagnosed on the bases of x-ray abnormalities or suggestive histology |
|  | Extra-pulmonary tuberculosis | TB involving organs other than the lungs e.g. pleura, lymph nodes, abdomen, genitourinary tract, skin, joints and bones, meninges etc. |
| Type of patient | New | Persons with TB who have never been treated for TB or have taken anti-TB drugs for less than one month |
|  | Previously treated | Persons with TB who have received one month or more of anti-TB drugs in the past |
| HIV status | HIV positive | Patient who had a positive HIV result at the time of TB diagnosis or during the course of treatment |
|  | HIV negative | Patient who had a negative HIV result at the time of TB diagnosis or during the course of TB treatment |
|  | HIV status unknown | Person who had no HIV test result |
| Directly observed therapy (DOT) plan | Facility-based | Taking of TB medication was monitored at the health facility by health care workers |
|  | Community-based | Taking of medication was monitored in the community by a community volunteer or relative |

Source: National Tuberculosis and Leprosy Control Program. Tuberculosis manual. Lusaka: Ministry of Health; 2017
