# Supplementary material for: Mortality among persons receiving tuberculosis treatment in Itezhi-Tezhi District of Zambia: A retrospective cohort study
Source: PLOS Glob Public Health. 2023 Feb 22;3(2):e0001234. doi: 10.1371/journal.pgph.0001234 (PMC10021721; doi:10.1371/journal.pgph.0001234)
Supplement: S1 Table — (DOCX) [file pgph.0001234.s003.docx]

**S1 Table: Sub analysis of characteristics associated with TB mortality in Itezhi-Tezhi District – among person with clinically diagnosed PTB (2015–2018), n=155**

| **Category** | **aHR (95% CI)** |
| --- | --- |
|  | **Multivariate** |
| Sex |  |
| Male | 1 |
| Female | 1.5 (0.2–3.0) |
| Age (years) |  |
| 0–14 | 1.0 (0.2–4.3) |
| 15–29 | 0.9 (0.4–2.1) |
| 30–44 | 1 |
| 45–59 | 0.8 (0.3–2.2) |
| 60 and older | 0.7 (0.2–2.3) |
| Type of patient |  |
| Retreatment | 1 |
| New | 2.2 (0.7–7.4) |
| DOT plan |  |
| Facility-based | 1 |
| Community-based | 2.2 (1.1–4.8)* |
| HIV status |  |
| Negative | 1 |
| Positive | 0.8 (0.4–1.8) |

**aHR, adjusted hazard ratio; CI, confidence interval; DOT, directly observed therapy; HIV, human immunodeficiency virus; TB, tuberculosis.**

***Significant at 0.20 level of significance.**
