# Supplementary material for: Mortality among persons receiving tuberculosis treatment in Itezhi-Tezhi District of Zambia: A retrospective cohort study
Source: PLOS Glob Public Health. 2023 Feb 22;3(2):e0001234. doi: 10.1371/journal.pgph.0001234 (PMC10021721; doi:10.1371/journal.pgph.0001234)
Supplement: S1 Fig — (DOCX) [file pgph.0001234.s005.docx]

**S1 Fig: Kaplan-Meier Survival curves by type of TB for persons with TB in Itezhi-Tezhi District (2015–2018), n=426.**

**Log-rank test**

| Observed Expected

TBType1 | events events

-------------+-------------------------

Clinical TB | 43 32.73

Confirmed TB | 28 38.27

-------------+-------------------------

Total | 71 71.00

chi2(1) = 6.60

Pr>chi2 = 0.0102
